# The DeepFish computer vision dataset for fish instance segmentation, classification, and size estimation

**DOI:** 10.1038/s41597-022-01416-0

**Published:** 2022-06-09

**Authors:** Nahuel Garcia-d’Urso, Alejandro Galan-Cuenca, Paula Pérez-Sánchez, Pau Climent-Pérez, Andres Fuster-Guillo, Jorge Azorin-Lopez, Marcelo Saval-Calvo, Juan Eduardo Guillén-Nieto, Gabriel Soler-Capdepón

**Affiliations:** 1 Department of Computing Technology, University of Alicante, Ctra. Sant Vicent, s/n, 03690 St. Vicent del Raspeig, Alicante Spain; 2 Instituto de Ecología Litoral, C. Sta. Teresa, 50, El Campello, 03560 Alicante Spain

**Keywords:** Computer science, Conservation biology

## Abstract

Preserving maritime ecosystems is a major concern for governments and administrations. Additionally, improving fishing industry processes, as well as that of fish markets, to have a more precise evaluation of the captures, will lead to a better control on the fish stocks. Many automated fish species classification and size estimation proposals have appeared in recent years, however, they require data to train and evaluate their performance. Furthermore, this data needs to be organized and labelled. This paper presents a dataset of images of fish trays from a local wholesale fish market. It includes pixel-wise (mask) labelled specimens, along with species information, and different size measurements. A total of 1,291 labelled images were collected, including 7,339 specimens of 59 different species (in 60 different class labels). This dataset can be of interest to evaluate the performance of novel fish instance segmentation and/or size estimation methods, which are key for systems aimed at the automated control of stocks exploitation, and therefore have a beneficial impact on fish populations in the long run.

## Abstract (summary)

**Table:** 

Measurement(s)	specimen size • fish species
Technology Type(s)	homography estimation • expert’s knowledge
Sample Characteristic - Organism	Mediterranean fish
Sample Characteristic - Environment	fish market
Sample Characteristic - Location	Levantine Balearic sea

## Background & Summary

Fisheries overexploitation is a problem in all oceans and seas globally. Authorities and administrations in charge of assigning quotas have very little fine-grained information on the fish captures, and instead use large-scale, coarse data to assess the health level of fisheries. Thus, being able to cross-match fish species and sizes, to the sea regions they were captured from, can be helpful in this regard, providing finer-grained information.

Previous attempts at assembling datasets for fish detection and classification exist, ranging from fish detection or counting in underwater images and video streams^1–3^, to counting on belts on trawler ships^4^, to classification in laboratory conditions^5,6^, or in underwater preprocessed images of single fish^7–9^, or single fish in free-form pictures^10^, as well as simultaneous detection and classification of several fish^11,12^. However, none of the works found in the literature addresses the topic of simultaneous instance segmentation and species classification, along with fish size estimation, in a fish market environment, as is the aim of this paper. Instance segmentation refers to the extraction of pixel-level masks for each individual object (in this case fish specimens), rather than bounding boxes (object detection), or class label masks (e.g. a single mask for all fish specimens of the same species, also referred to as semantic segmentation). Moreover, works in the literature use pictures taken in laboratory conditions (with a single fish per image, shown from the side), or in underwater conditions. Only French *et al*.^4^ uses pictures of fish catches on a belt, for counting purposes. Table 1 shows a summary of the datasets identified in the literature, along with their characteristics, including how the proposed dataset compares.

**Table 1:** Summary of previous datasets found in the literature, and comparison to proposed dataset.

Dataset	Images (instances)	Categories	Aim of dataset
Spampinato *et al*.^5^	360 (360)	10	classification
Ogunlana *et al*.^6^	150 (150)	2	classification
Cutter *et al*.^1^	929 (1,005)	—	detection (underwater)
French *et al*.^4^	443 (–)	—	counting (on trawler belts)
Sung *et al*.^2^	video data	—	detection (underwater)
fish4Knowledge^11^	2.5·10^6^ to 16·10^6^ fish	24	detection, classification (underwater)
NCFM^10^	—	8	classification
Chhabra *et al*.^8^	435 (435)	8	classification (underwater)
Alsmadi *et al*.^9^	400 (400)	24	classification
Fish-Pak^7^	915 (915)	6	classification
Zhang *et al*.^3^	1,501 (150k)	—	detection, counting
Brackish dataset^12^	89 videos	6	detection, classification
URPC^18^	5,543 (41,441)	5	classification (underwater)
DeepFish *(Proposed)*	1,291 (7,339)	59	instance segmentation, classification, size estimation (at fish market)

The ground truth available for each is limited by the ‘aim of the dataset’ (i.e. classification datasets do not provide pixel-wise masks).

**Fig. 1:**
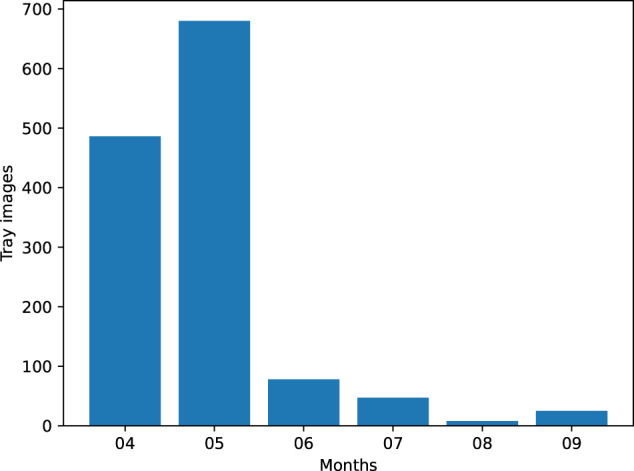
Temporal distribution of fish tray images captured. It can be observed that April (04) and May (05) were much more active than the rest of months. This is due to several contributing factors.

The DeepFish project (website: http://deepfish.dtic.ua.es/) is aimed at providing fish species classification and size estimation for fish specimens arriving at fish markets, both for the automation of fish sales, and the retrieval of fine-grained information about the health of fisheries. For a period of six months (April to September 2021), images have been captured at the fish market in El Campello (Alicante, Spain). Images of market trays show a variety of fish species, including targeted as well as accidental captures from the ‘Cabo de la Huerta’, an important site for protection and preservation of marine habitats and biodiversity as defined by the European Comission Habitats Directive (92/43/EEC). From the pictures, a total of 59 different species are identified with 12 species having more than 100 specimens and 25 with more than 10 specimens, as shown in Table 2. There is a high imbalance of species captured due to the natural variation in fish species populations according to seasonality and other ecological factors (rarity of the species, i.e. total population count, etc). Due to some species showing sexual dimorphism (i.e. *Symphodus tinca*), this species is split into two separate class labels, leading to a different number of species, and class labels (59 species, but 60 class labels). The dataset presents a high temporal imbalance too. As shown in Fig. 1, the capture of new fish tray images was not evenly distributed during the six month study period. Several factors contributed to this: wholesale fish market operating days (e.g. no weekend data, holidays and stop periods, etc.), fish species variability (one of the aims was to be able to capture at least 100 specimens from several species, and seasonality meant some could not be available for capture in later months), as well as the time availability of research group members to attend the fish arrival, tray preparation and auctioning in the evenings.

**Table 2:** Distribution of fish species in the dataset.

Species (scientific name)	Count	Species (scientific name)	Count
*Mullus surmuletus*	**1245**	*Dentex dentex*	88
*Serranus scriba*	**1104**	*Merlucius merlucius*	56
*Pagellus acarne*	**901**	*Diplodus vulgaris*	42
*Diplodus annularis*	**793**	*Sardinella aurita*	36
*Pagellus erythrinus*	**637**	*Serranus cabrilla*	28
*Spicara mæna*	**636**	*Sparus aurata*	22
*Pagrus pagrus*	**585**	*Sarda sarda*	21
*Mullus barbatus*	**546**	*Scorpæna notata*	18
*Symphodus tinca* (335, 211)	**546**	*Symphodus mediterraneus*	16
*Sepia officinalis*	**265**	*Muræna helena*	14
*Scorpæna porcus*	**263**	*Lithognathus mormyrus*	11
*Sphyræna sphyræna*	**126**	*Raja radula*	11
*Diplodus sargus*	95	Other 34 species (≤10 each)	140

A total of 7,339 fish specimens were labelled from 59 different species. Since *Symphodus tinca* shows sexual dimorphism, 60 different class labels are considered, with additional classes for male (♂) and female (♀) specimens. Only species above 10 specimens are listed here (25 species; in 26 labels). All other 34 species have 10 or fewer specimens and are shown grouped here.

**Fig. 2:**
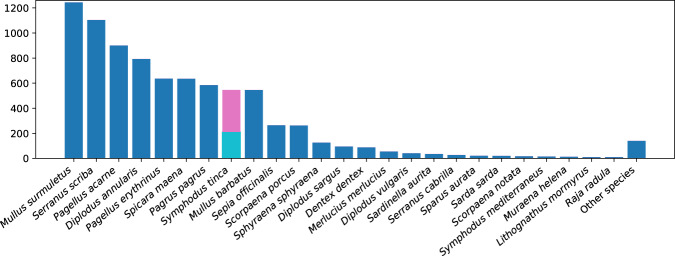
Graphical view of the distribution of fish species in the DeepFish dataset for species above 10 specimens. Note, *Symphodus tinca* is considered separately due to sexual dimorphism (211 male; 335 female samples).

**Fig. 3:**
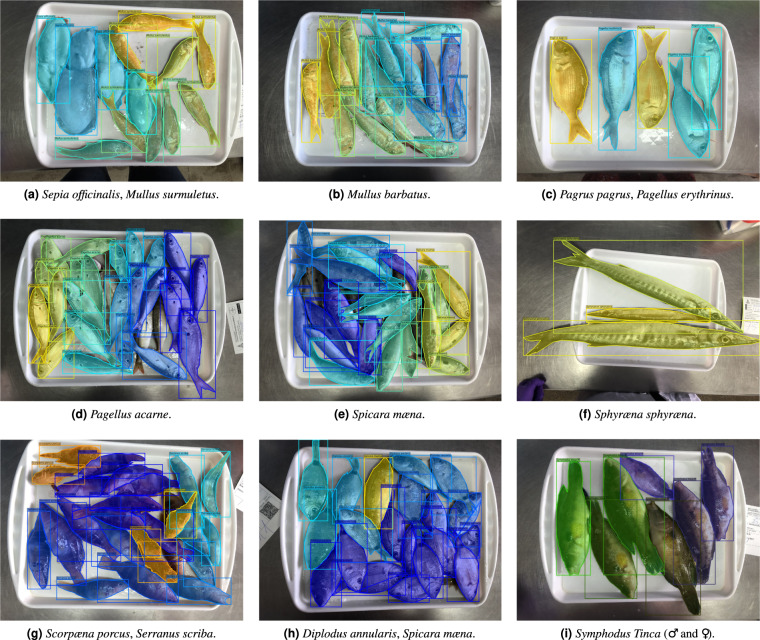
Examples of ground truth fish instance masks with class labelling, showing the 12 species (13 labels) with more than 100 specimens (in bold in Table 2).

The resulting DeepFish dataset introduced here contains annotated images from 1,291 fish market trays, with a total of 7,339 specimens (individual fish instances) which were labelled (species and mask) using a specially-adapted version of the *Django labeller* instance segmentation labelling tool^13^. Subsequently, another JSON file is generated, following the Microsoft Common Objects in Context (MS COCO) dataset format^14^, which can be directly fed to a neural network. This is done via a script that is also provided^15^. Figure 2 shows the distribution of individuals for the selected species within the dataset. Furthermore, Fig. 3 shows examples of the trays, with instance segmentation (ground truth silhouette, i.e. as an interpolation from human-provided points) along with species labelling (different colour shading).

From the point of view of research, this data is important for the classification of fish species, instance segmentation, as well as specimen size estimation (e.g. as a regression problem, or otherwise). From an end-results perspective, data *automatically* labelled with fish instance segmentation accompanied by species name and estimated size is useful to different stakeholders, namely: fishing authorities (to understand how much of each species is being caught per zone), maritime conservation (to calculate depletion of fisheries), but also managers of the markets themselves, as well as clients (digitized sales, e-commerce), etc.

The usage of the provided data can be manifold, as it can be used for several problems, namely: object detection and classification, which involves finding objects (in this case fish specimens) providing a bounding box, and a class for each of these boxes; additionally, the data can also be used for semantic segmentation, which can provide a pixel-wise segmentation of the image providing labels (in this case species labels) to different pixel regions of the image; furthermore, also instance segmentation is possible, in which not just a single label for all instances of the same species is provided, but each specimen is provided with a mask (specimen segmentation), as well as a label (species). Furthermore, several measurements of each fish are provided, which can also be used to estimate their size, since they have been shown to be correlated with each other^16^. These are estimated from the calculated homography (given the tray size is known), given the burden of measuring each fish due to the large amount of specimens in the dataset.

## Methods

Data acquisition was performed on a mobile phone without modifications, specifically an iPhone 8 model, from Apple Inc. The requirement was that the image had to be captured horizontally, with the phone as parallel to the tray as possible (i.e. shot perpendicular to the fish tray), and with a fully visible tray. Furthermore, another requirement was to aim for a minimum of 1,200 images as the target to have enough data for any subsequent model training.

A time frame of 6 months was considered, to account for species variability due to seasonality of fish captures. This led to the 60 class labels mentioned before, on a total of 1,320 pictures of trays containing at least one species of interest. The species were selected based on their frequency of appearance in the trays at the fish market (total counts) as well as their commercial interest (fish species that are common in artisanal fishing culture and cuisine locally). Of all the images collected, 29 were left unannotated, but are provided nonetheless. The main reason is that the images were of lower quality (e.g. out of focus; too tilted, i.e. causing bad perspective view, and similar issues).

Django labeller^13^ was then used to label the images, by adapting it to the specifics of the problem, that is, by including a list of species labels and allowing for four different size specification (eye diameter, width at waist, length to tail, and total length) all provided to millimetre accuracy, per instance. These four measurements have been taken considering several factors: 1) eye diameter of fish has been correlated^16^ with total fish length, therefore, if at least the eye is visible in full, even in the presence of partial body occlusions, the total size of the fish specimen can still be inferred; 2) as in the previous case, the width at the waist can also be correlated to the total length when the fish body length is not fully visible, but the widest part is; 3) in many species, specially in the *Thunnus* genus, the tail is fragile and can easily bruise and break, therefore a measurement to the tail base is common, this is often referred to as ‘standard measure’, when compared to the ‘total measure’ when the tail is intact and accounted for. From the manual human expert labelling, a JSON file for each tray is generated, including pixel-level instance segmentation for each fish; its species; and its different size parameters, as just explained. Finally, a Python script^15^ is used to convert between Django labeller JSON format, and MS COCO JSON format, which is widely accepted by many neural networks for training.

## Data Records

The data is openly available to the public, in a Zenodo repository^17^. The files that make up the dataset are the 1,320 JPEG images of fish trays; and, additionally, the 1,291 JSON files that accompany most of the images, as annotations (in Django labeller format). All files follow a naming convention, as follows:

- JPEG images: <DD>_<MM>_<YY>-B(.) <NN>.jpg, for instance: 7_06_21-B7.jpg or 13_04_21-B.18.jpg, and
- JSON annotations: <DD>_<MM>_<YY>-B(.) <NN>__labels.json, for instance: 7_06_21-B7__labels.json or 13_04_21-B.18__labels.json,

where <DD> stands for the day, <MM> month, and year <YY>, respectively; and the letter ‘B’ stands for *batch* (i.e. each tray), accompanied by <NN> tray number within that date. Please note, that an optional dot can follow the ‘B’ in the name (i.e. ‘B.’), in some files.

## Technical Validation

**Fig. 4:**
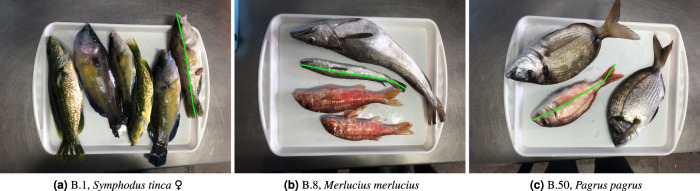
Images from the trays showing the specimens used as samples in Table 3. The ground truth ‘total size’ used for size estimation from homography is shown as green line segments.

**Fig. 5:**
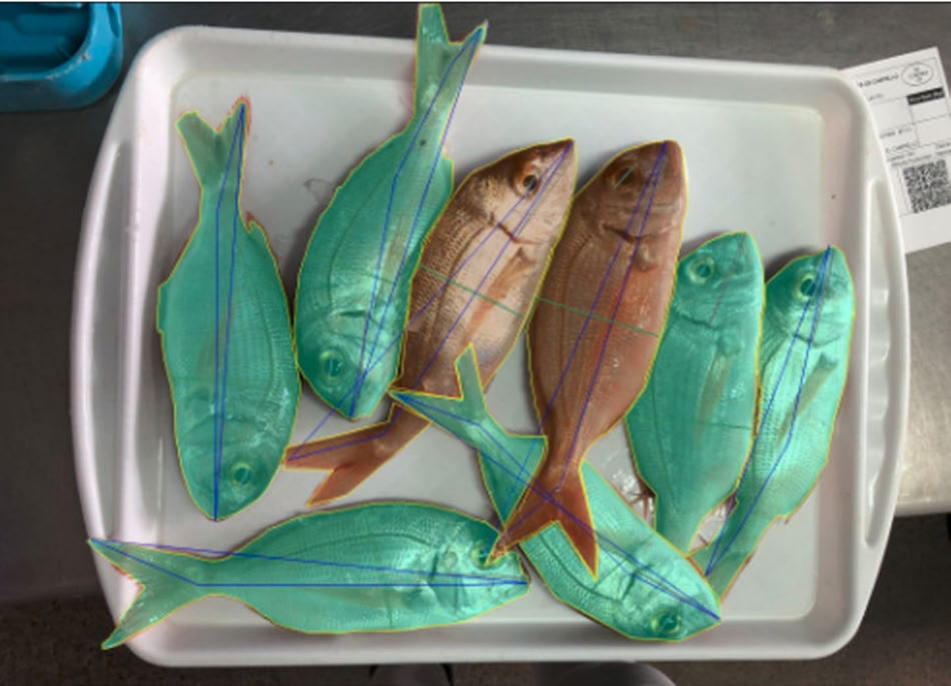
Example of provided instance segmentation ground truth, including species labelling. Different measurements of fish size are provided too (as depicted): diameter of the eye, width at the waist, and two different length measurements (including, or excluding the tail).

With regard to the technical validation of the data, a team of two marine biologist experts was in charge of species labelling. First, each biologist would assess the species of fishes present in a tray, and label them accordingly; then, the data was cross-checked by the other expert. A similar procedure was taken for fish measurements. However, due to the large volume of fish specimens to label, individual sizes were automatically derived from the calculated image homography, using the size of the tray (which is known). To validate this approach, a small subset of specimens was taken and physically measured by the expert team using an ictiometer (a fish-measuring device). When compared to the actual measurements, the homography-derived sizes show an average error of around 2 to 3%. This is shown in Table 3, that presents some samples for illustration, along with total and relative errors. Figure 4 shows the ground truth size of fish specimens used as samples in Table 3. Furthermore, as explained, four different measurements were annotated for each specimen: eye diameter, width at the waist, length to the tail base (a.k.a. ‘standard’ length), and total length. The eye diameter and width at the waist can be used to derive standard and total lengths, in case of partial occlusion by another fish, as explained by Richardson *et al*.^16^. Figure 5 shows examples of the size labelling provided (all four measurements).

**Table 3:** Samples of fish specimens with their actual size, and inferred size using homography estimation.

Tray, specimen	Specimen size (cm)	Estimated size (cm)	Error (cm)	Relative Error (%)
B.1, *Symphodus tinca*	25.3	25.64	0.34	1.33%
B.8, *Merlucius merlucius*	25.0	24.29	0.71	2.85%
B.50, *Pagrus pagrus*	24.0	23.05	0.95	4.00%
Mean error			0.66	2.73%

Errors are provided in centimetres and as a relative error (in percentage).

## Usage Notes

For faster, more convenient download, all image files are provided bundled in several compressed ZIP files named as fish_tray_images_<YYYY>_<MM>_<DD>.zip. Similarly, individual JSON files are also bundled into a single compressed ZIP file named fish_tray_json_labels.zip.

Once the files are downloaded, and in case the user wants to use them in a deep neural network, or other machine learning model that accepts the MS COCO format, the accompanying script^15^ can be used, to generate train and validation sets, as required. However, for convenience, the labelling JSON files are also offered pre-converted to the COCO format, in which all previous files are aggregated into a single additional file named coco_format_fish_data.json in the repository.

## Data Availability

The images and Django labeller annotation files (JSON) are available on the mentioned Zenodo repository^17^. Furthermore, the code to obtain JSON files in the MS COCO format is published alongside this dataset, and is made available online^15^.
